# Lie symmetry analysis of the effects of urban infrastructures on residential property values

**DOI:** 10.1371/journal.pone.0255233

**Published:** 2021-08-05

**Authors:** Chien-Wen Lin, Jen-Cheng Wang, Bo-Yan Zhong, Joe-Air Jiang, Ya-Fen Wu, Shao-Wei Leu, Tzer-En Nee

**Affiliations:** 1 Department of Electrical Engineering, National Taiwan Ocean University, Keelung City, Taiwan, Republic of China; 2 Department of Computer Science, National Taipei University of Education, Taipei City, Taiwan, Republic of China; 3 Department of Biomechatronics Engineering, National Taiwan University, Taipei City, Taiwan, Republic of China; 4 Department of Electronic Engineering, Chang Gung University, Tao-Yuan City, Taiwan, Republic of China; 5 Department of Medical Research, China Medical University Hospital, China Medical University, Taichung City, Taiwan, Republic of China; 6 Department of Electronic Engineering, Ming Chi University of Technology, New Taipei City, Taiwan, Republic of China; 7 Artificial Intelligence Research Center, Chang Gung University, Tao-Yuan City, Taiwan, Republic of China; China University of Mining and Technology, CHINA

## Abstract

Due to the complexity of socio-economic-related issues, people thought of housing market as a chaotic nucleus situated at the intersection of neighboring sciences. It has been known that the dependence of house features on the residential property value can be estimated employing the well-established hedonic regression analysis method in teams of location characteristic, neighborhood characteristic and structure characteristic. However, to further assess the roles of urban infrastructures in housing markets, we proposed a new kind of volatility measure for house prices utilizing the Lie symmetry analysis of quantum theory based on Schrödinger equation, mainly focusing on the effects of transportation systems and public parks on residential property values. Based on the municipal open government data regularly collected for four cities, including Boston, Milwaukee, Taipei and Tokyo, and all spatial sampling sites were featured by United States Geological Survey (USGS) National Map, transportation and park were modelled as perturbations to the quantum states generated by the feature space in response to the environmental amenities with different spatial extents. In an attempt to ascertain the intrinsic impact of the location-dependent price information obtained, the similarity functions associated with the Schrödinger equation were considered to facilitate revealing the city amenities capitalizing into house prices. By examining the spatial spillover phenomena of house prices in the four cities investigated, it was found that the mass transit systems and the public green lands possessed the infinitesimal generators of Lie point symmetries Y_2_ and Y_5_, respectively. Compared statistically with the common performance criteria, including mean absolute error (MAE), mean squared error (MSE) and, root mean squared error (RMSE) obtained by hedonic pricing model, the Lie symmetry analysis of the Schrödinger equation approach developed herein was successfully carried out. The invariant-theoretical characterizations of economics-related phenomena are consonant with the observed residential property values of the cities internationally, ultimately leading to develop a new perspective in the global financial architecture.

## Introduction

Big data through the internet providing ubiquitous access to natural and human information is rapidly expanding to all realms of the complex systems such as urban and real estate economics [1–5]. It may transpire that an increasing number of physicists have adopted a complex system to approach in analyzing and modeling the financial and economic systems, as most experimental scientists are wont to do [6, 7]. Based on the general assumption of homogeneity of the housing product, e.g., perfect market operations, the well-established hedonic pricing method (HPM) has deployed regression analysis to explore key impact on the basis of the relationship between the independent and dependent variables. The independent variables are the individual structures, the neighborhood, the environment and house age, etc., while the dependent variable is the house price, indicating that housing-market price is a function of tangible characteristics, building characteristics and influencing factors [8, 9]. Resulting in low values of the mean absolute error (MAE), mean squared error (MSE) and, root mean squared error (RMSE), it has been widely acknowledged that the HPM successfully evaluated the influencing factors of house price markets in most individual cities [10–14]. However, nationwide’s house price review of both formal literature and informal sources for different cities in different counties has revealed that the processes of data extraction had hitherto been plagued with challenges, such as the variability in the standardized metrics of dataset quality, the barriers of language and idiosyncratic feature of the city [15–20]. Accordingly, the time-consuming task for data homogenization effort should be approached in a very meticulous manner. The scope and aspect of methodologies and techniques used by city-level studies may be employed only in the specific city, and not be applicable to all metropolitan areas around the globe.

In response to systemize the public dataset for attaining a reasonable level of mathematical sophistication linking directly with various fields of natural and socio-economic science, researches in feature engineering are burgeoning with academic and industry groups. Based on data science with powerful cross-disciplinary technologies, including machine learning, probability and statistics, abstract algebra and differential equation, in big data analytics, characterization methods in the values of residential properties have became vital parts in the progress for assessing and interpreting the effects of urban infrastructures on house prices [21–23]. On the other hand, many attempts have been already made for applying the principles of fundamental physics, i.e., classical mechanics, electrodynamics, statistical mechanics and quantum mechanics, to estimate the dependence of environmental amenity feature on the residential property values in the so-called ecophysics or financial engineering regimes [24–29]. The demand potential model was constructed by combining the main ideas of classical electrodynamics and standard economics [30]. Using partial-integro differential equations (PIDE), jump-diffusion models were proposed to describe the house price evolution in probability space [31]. Furthermore, based on the simulation of stochastic processes by diffusion equations coupled to stochastic sources, many mathematical methods, such as the Hamiltonians and Feynman path integral formulation, were fascinatingly involved in the analysis of financial systems [29, 32]. It is possible to derive a new kind of volatility measure for house prices utilizing the quantum mechanics widely based on Schrödinger equations as well [33–35]. Taking into account for house prices and price changes, Bohmian model for behaviors of the financial market has been developed in teams of information perturbation of Hamiltonian equations [35, 36].

Nevertheless, as a matter of fact, modern theoretical studies suggest that symmetry is a foundational principle of interdisciplinary science [37]. In order to capture the salient features, group theory has thus considerably presented a framework to explore symmetries in the observed or measured information and to turn the large and complex data into knowledge and intelligence. The existence of a symmetry in a house price system means that it is possible to extract invariances from monitoring data to profile physical systems. Applying the group action on the feature spaces, the intrinsic structures from which the data is drawn are conserved [37]. Moreover, multi-dimensional datasets, e.g., the so-called big data, can be decomposed into lower-dimensional datasets, e.g., the so-called small data, while both datasets are homomorphic in aspect of the invariance properties. Mathematically, by virtue of the automorphism, the group actions determined by the choice of regional house price measurements leave all structural relations undisturbed globally [38]. Consequently, in many practical situations, close inspection of the symmetry characteristics of the relatively small data can immediately provide the scientists concerned with a better accuracy of the statistical analysis from big data. Group theory brings in a new aspect to the development of the housing market information system in every country [37, 38]. However, effective group theory approach of residential property market is still lack. Methods based on the framework of symmetry and conservation law analysis can be systematically applied to wide classes of partial differential equation and ordinary differential equation models, which needs to be paid more attention by socio-economic scientists, computer engineers, mathematicians, etc [39, 40]. The recent advances in nonlinear integrable systems may open a new route in the search for the integrability and analytical methods of high-order nonlinear differential equation, symmetric equations and discrete equations, including the transformation relationship between equations, the construction of integrable clusters, symmetry and conservation laws, soliton solutions and quasi-periodic wave solutions and integrable properties [41–45]. Inescapably, the nonlocal symmetry method for many nonlinear systems has been successfully studied by using the truncated Painlevé method and the Möbious (conformal) invariant form [46, 47].

Toward this end, in the present work, the symmetry-based strategy to assess the roles of urban infrastructures in housing markets was made possible by adopting the approach of Lie point symmetries admitted by Schrödinger equation, mainly focusing on the effects of transportation systems and public parks on residential property values. All official house price datasets comprise of tabular data for four major cities, including Boston, Milwaukee, Taipei and Tokyo, gathered from the municipal open government data platforms in the countries. The location of public transit stops and park characteristics were obtained from United States Geological Survey (USGS) National Map (USGS National Map) [48]. The Lie analysis approach (LSA) based on the Schrödinger algebra, i.e., Lie point symmetries of Schrödinger equation, was contrived to elucidate the correspondence between Lie symmetry and the influence factors for the housing prices. Transportation and open-space were both thought as quantum perturbations with different spatial extents. Accordingly, in order to facilitate the city amenities capitalizing into house prices, the similarity functions associated with the Schrödinger equation were proposed in an attempt to ascertain the impact of the location-dependent price information obtained. The validity of the invariant-theoretical characterizations of economics-related phenomena was corroborated by the comparison with the common performance criteria obtained by hedonic pricing model (HPM). The findings concerning the invariant effects of the symmetry law on the house price dynamics could provide a new paradigm for understanding how the group-theory approach governs the fluctuating price behaviors of the worldwide housing markets.

## Methods and datasets

**Fig 1:**
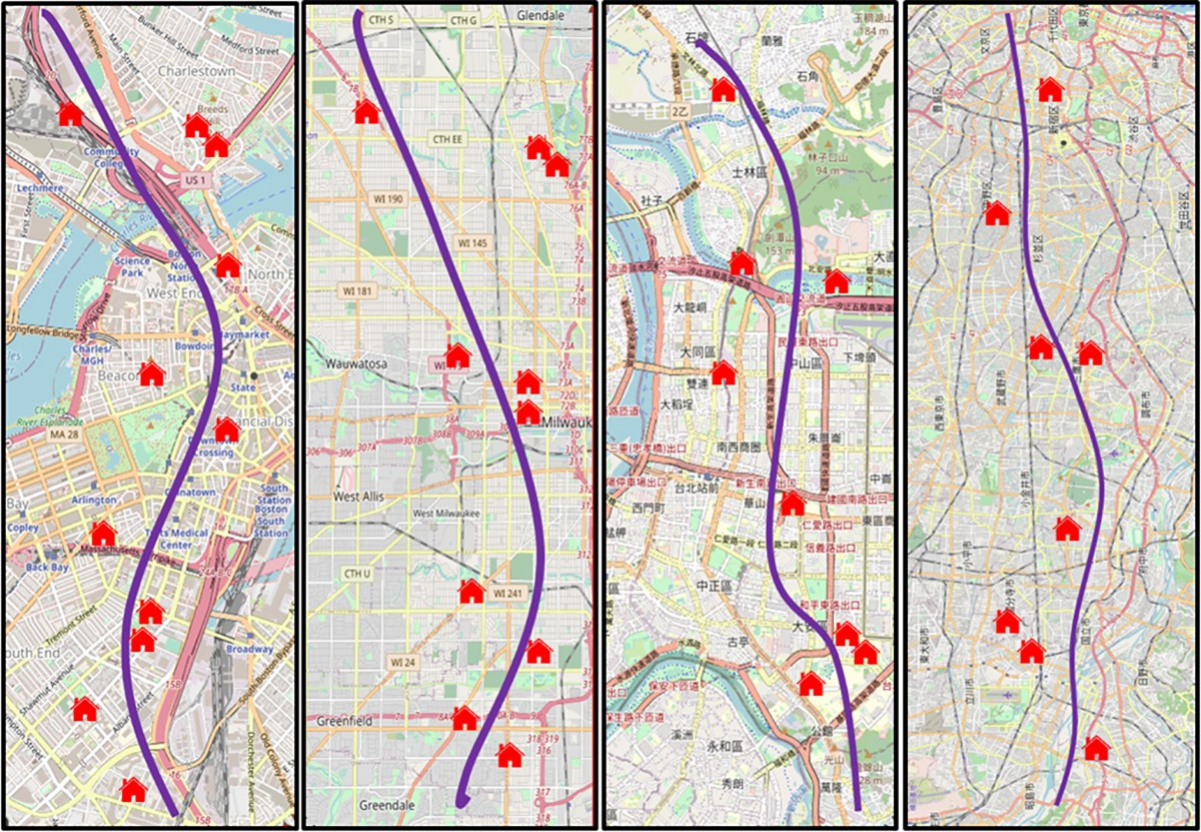
The selected routes in (a) Boston; (b) Milwaukee, (c) Taipei; and (d) Tokyo.

Fig 1 made of information from USGS National Map Viewer was a schematic diagram of routes selected for the symmetry analysis of the cities of Boston, Milwaukee, Taipei, and Tokyo, respectively. The choice of route for examining housing price mainly depended on the influencing factors, i.e., a station, a park or both, deemed to be analyzed and explored further. For reasons of visual brevity, only a few samples of the map legends, i.e., Houses, which appeared in USGS National Map on the relative location were schematically deposited in the figure.

**Fig 2:**
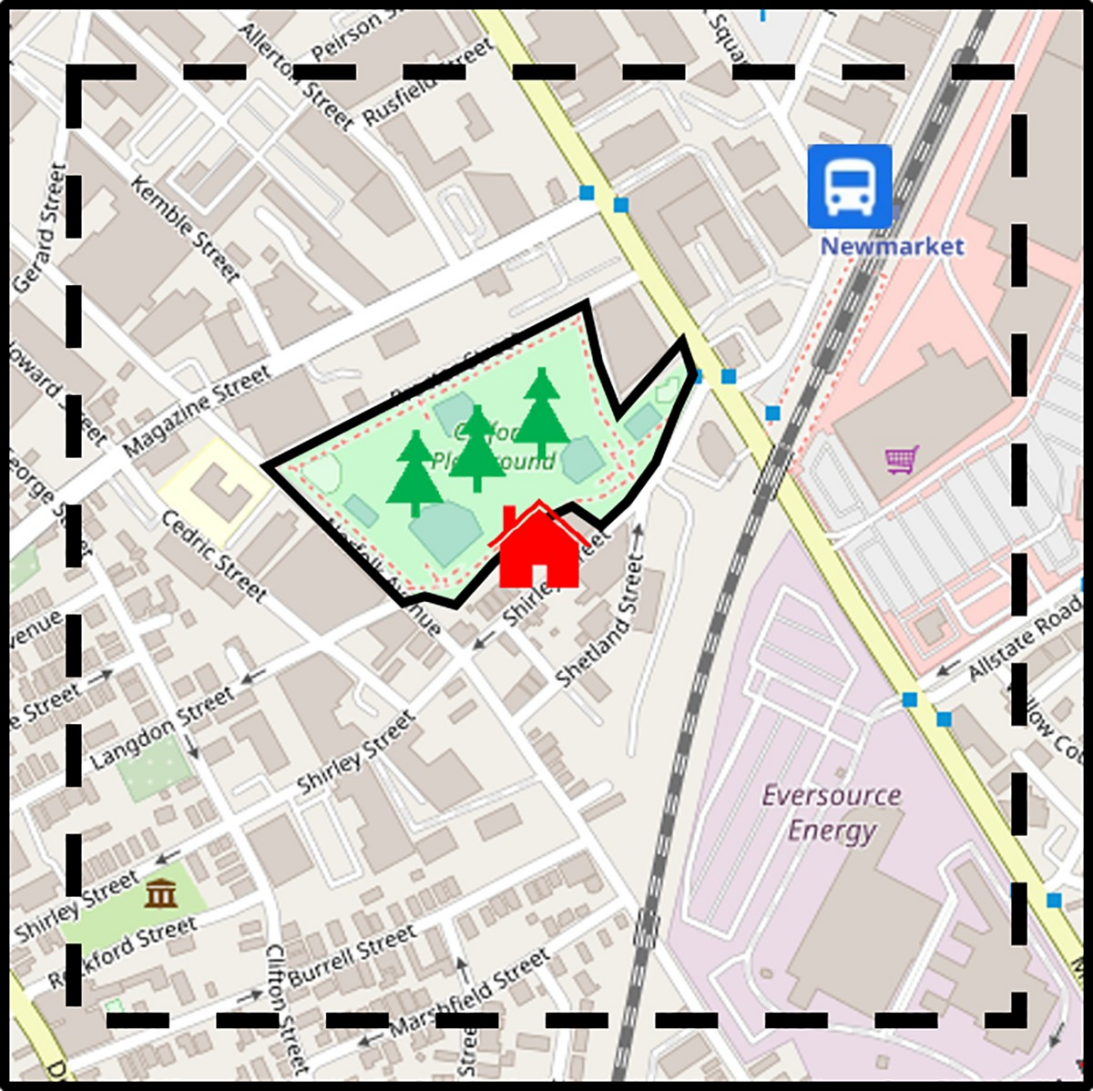
Schematic diagram of the definitions of park size and distance to transportation.

As similar to those which were previously reported in many other public amenities, we modified the objective measurable attributes affecting property price to account for park size and distance to transportation, as schematically illustrated in Fig 2 [19, 40, 49–51]. To analyze the percentage of green area, we placed the house of interest at the center of a square parcel with sides parallel to the North-South direction and East-West direction on the map, while the direct distance to transportation, was found by using USGS National Map. This study adopted the real property price datasets based on freely downloadable data from the local government institute websites of the cities of interest [52–55].

HPM employed in this work was a statistical regression model in determining a housing price. Based on the well-known machine learning approach, HPM estimated the relationship between the housing prices and the features for the four cities, investigated was given by *y* = *ax*_1_+*bx*_2_+*cx*_3_+…+*nx*_*i*_ where *y* was the housing price, *x*_0_ was the intercept term, *x*_1_,*x*_2_,*x*_3_,…*and x*_*i*_ were the attributes of a house, and a, b, c,…and n were the correlation coefficients of the features studied [14, 56].

**Fig 3:**
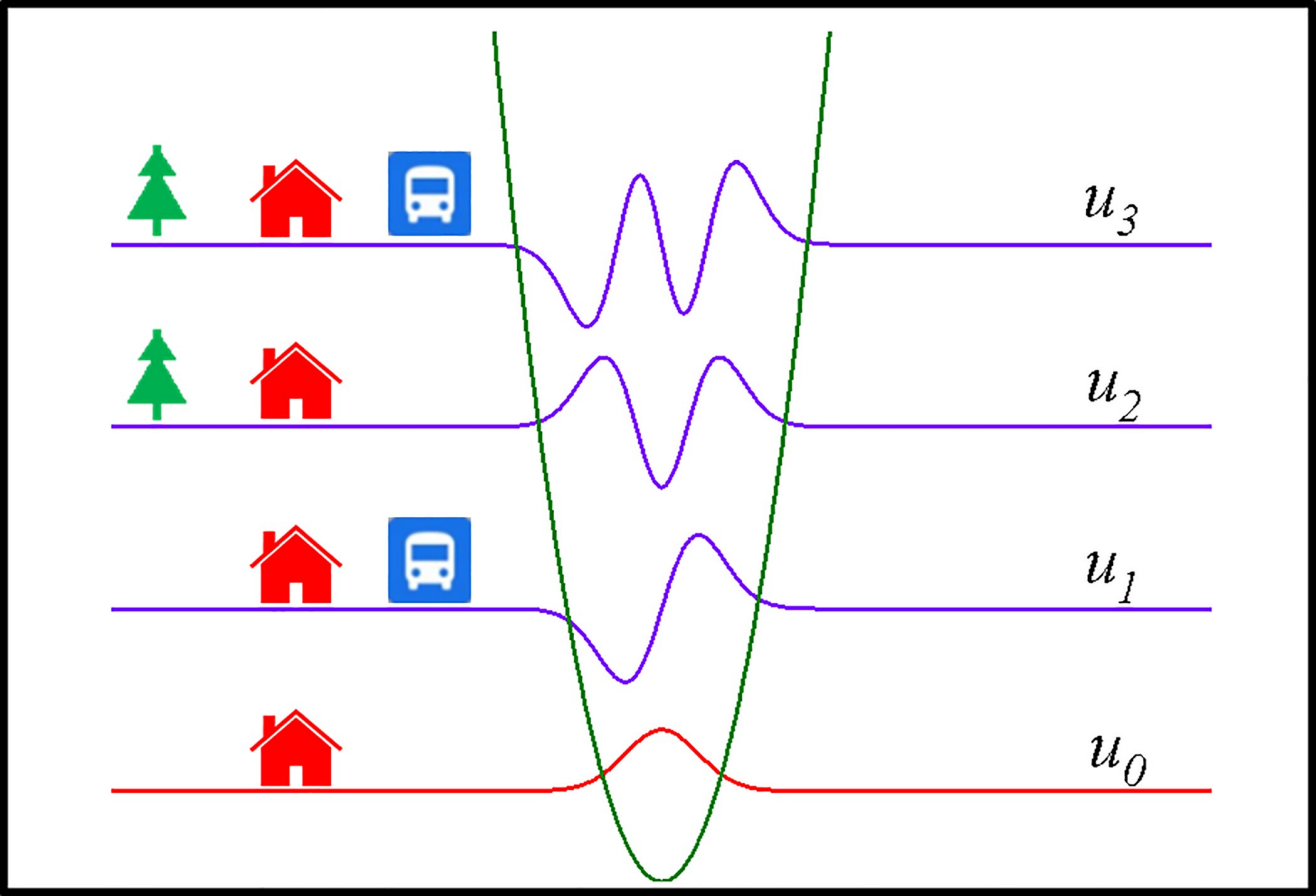
Schematic plot of HPW in the quantum mechanical harmonic potential.

Due to the complexity of socio-economic-related issues, people thought of housing market as a chaotic nucleus situated at the intersection of neighboring sciences. As usual, the changes in property value were statistically subject to nonlinear stochastic equations [57]. Generally, housing characteristics corresponding to internal, external and capitalist features used in the estimation of the HPM were categorized thematically into three fundamental types, including location, structure and neighborhood factors [58, 59]. In this work, we treated the house price volatility and city infrastructure as represented by a dynamical wave and a continuous field, respectively, not explicable in the current framework of socio-economic sciences, which were subject to Schrödinger equations. Accordingly, the urban housing market system was phenomenologically understood as a crystal-like complex system with an arrangement of potential wells or barriers, where structure and finance factors were regarded as potential wells and barriers. This approach was conceptually similar to the so-called gravity model or potential mode [60]. The house price may be viewed with great interest as a socio-economic wave-packet, and hereafter we called it housing price wave (HPW), as schematically illustrated in Fig 3.

The observed regularities in the evolution of prices in the housing market system was thus provided by quantum effects on a macroscopic scale. Furthermore, external features, such as accessible and inaccessible infrastructures, were then treated as perturbations on the original Hamiltonian, giving rise to a change of the property value. As far as the modulation of spatial structures of the HPW was concerned, transportation systems and public parks were generally deemed to be positive influencing factors, leading to the changes including both the wave packet shape and extension of the spatial envelope. The absolute target values, i.e., actual house price, were undoubtedly considered difficult to be predicted. Because of that the exact conditions for many features were not adequately described and the properties of a location-related factors in reality were very complex, it was difficult to model these known or unknown features. Based on the well-established Lie point symmetries, Schrödinger equation with quantum mechanical harmonic potential in one space dimension has seven nontrivial the infinitesimal generators of Lie point symmetries or so-called Lie-Bäcklund infinitesimal generators, i.e., $Y_{1}=e^{it}\frac{\partial }{\partial x}-ux\frac{\partial }{\partial u}$, $Y_{2}=e^{-it}\frac{\partial }{\partial x}+ux\frac{\partial }{\partial u}$, $Y_{3}=\frac{\partial }{\partial t}$, $Y_{4}=e^{i2t}[\frac{\partial }{\partial t}+ix\frac{\partial }{\partial x}-iu(\frac{1}{2}+x^{2})\frac{\partial }{\partial u}]$, $Y_{5}=e^{-i2t}[\frac{\partial }{\partial t}+ix\frac{\partial }{\partial x}+iu(\frac{1}{2}-x^{2})\frac{\partial }{\partial u}]$, $Y_{6}=u\frac{\partial }{\partial u}$ and $Y_{7}=\phi (t,x)\frac{\partial }{\partial u}$, where *u* and *ϕ*(*t*,*x*) were an invariant solution and any solution of Schrödinger equation, respectively [61]. The invariant solutions associated with the Lie symmetry approach (LSA) were candidates for the generation of HPW functions under influence of the perturbed harmonic potentials corresponding to transportation and open-space located on the USGS National Map. The time-independent invariant solutions denoted by $u_{1}(x)=2(x-x_{1})e^{-\frac{1}{2}k_{1}{\left(x-x_{1}\right)}^{2}},\;u_{2}(x)={[4{\left(x-x_{2}\right)}^{2}-2]e}^{-\frac{1}{2}k_{2}{\left(x-x_{2}\right)}^{2}}$, and $u_{3}(x)={[8{\left(x-x_{3}\right)}^{3}-12(x-x_{3})]e}^{-\frac{1}{2}k_{3}{\left(x-x_{3}\right)}^{2}}$ corresponding to Station, Park and both incorporations in new building, respectively, while $u_{0}(x)=e^{-\frac{1}{2}k_{o}{\left(x-x_{0}\right)}^{2}}$ denoted a new building as schematically illustrated in Fig 3, where u_i_ and k_i_ were location shifts and spatial spreading parameters for i = 1,2,3, respectvely [61]. These wave-related descriptions are similar to the information fields proposed by Olga Choustova [35]. It can be also characterized that the spatial broadening of u_3_(x) was larger than that of u_2_(x), while that of u_1_(x) was the smallest among these three invariant solutions.

This article uses mean absolute error (MAE), mean squared error (MSE), root mean squared error (RMSE) and to evaluate HPM and LSA. MAE, MSE and RMSE values can be calculated using $MAE=\frac{\sum_{i=1}^{n}\left|P_{i}-y_{i}\right|}{n},\;MSE=\frac{\sum_{i=1}^{n}{\left(P_{i}-y_{i}\right)}^{2}}{n}$ and $RMSE=\sqrt{\frac{\sum_{i=1}^{n}{\left(P_{i}-y_{i}\right)}^{2}}{n}}$, respectively, where *P*_*i*_ was the transaction value obtained from the datasets and *y*_*i*_ was the value after the self-built point from the model of interest. The low values of errors demonstrated the high precision of the model used.

## Results and discussion

Given the concerns with the homogeneity of the housing product, e.g., perfect market operations, the hedonic model served as the machine learning basis in this work for characterizing the effects of urban infrastructures on residential house values at individual property level. To begin with, property prices only in the residential district of Boston were examined employing the well-developed HPM. Subsequently, the HPM can be extended to other three cities, i.e., Milwaukee, Taipei and Tokyo, for systematic analyses.

Table 1 presented the dataset description with the mean, median and standard deviation computed for ten feature variables surveyed, including eight internal structure characteristics and two external characteristics [59, 62]. Resulting from the comparison of the median and the mean for the variables in Table 1, the samples collected might be considered to obey normal distribution, essentially providing unbiased baselines to characterize and understand the effect of the features of interest on housing prices in Boston.

**Table 1:** Descriptive statistics of the data for Boston.

Variables	Definition	Mean	Median	Standard Deviation
LAV_TOTAL	The total assessed value, in natural logarithm.	13.7361	13.6533	0.5799
LLAND_SF	The parcel’s land area of residential property, in natural logarithm.	7.6093	7.4967	0.5656
LAGE	The age of residential property, in natural logarithm.	4.6181	4.8040	0.7168
LR_T_RMS	The total number of rooms, in natural logarithm.	2.2848	2.3026	0.3855
LR_BDRMS	The total number of bedrooms, in natural logarithm.	1.4876	1.3863	0.3813
LN_FLOORS	The levels in the structure located, in natural logarithm.	0.9656	1.0986	0.2260
R_KITCH	The total number of kitchens.	1.8571	2.0000	0.8416
R_FULL_BTH	The total number of full baths.	2.4082	2.0000	1.0591
R_HALF_BTH	The total number of half baths.	0.4694	0.0000	0.6158
GREEN	The unit rectangular green area percentage.	4.5397	3.9100	2.6531
TRAFFIC	The direct distance to the nearest station.	5.6059	5.5581	2.3905

**Fig 4:**
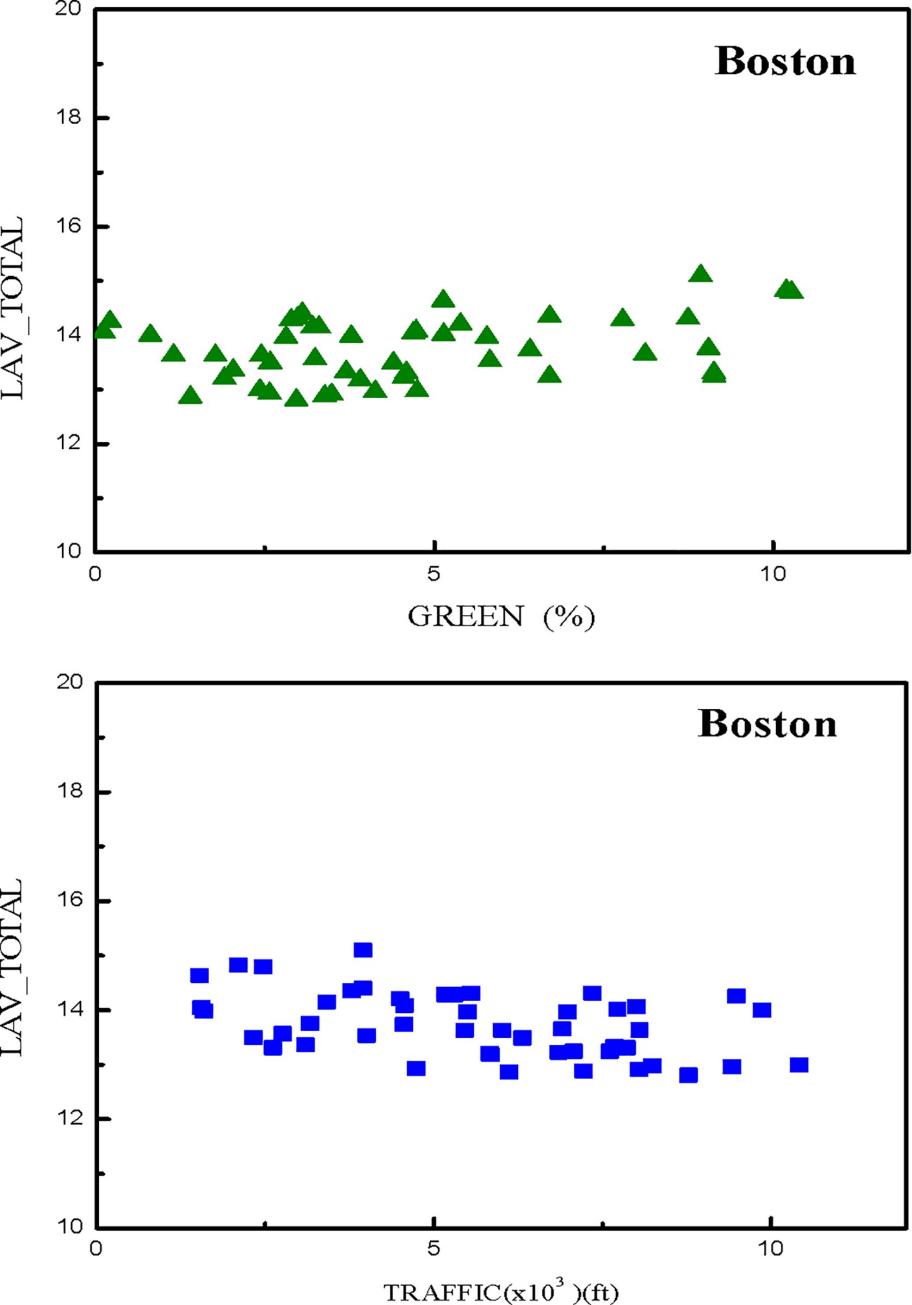
Data visualization to portray the relationships between property prices and selected features for (a) the green area percentage within a square unit; (b) the direct distance to the nearest station.

In an attempt to assess the possibility of applying principal component analysis, a correlation matrix that accounted for correlations among the features observed in Boston was used to evaluate the 121 dependency between multiple variables simultaneously, as shown in Table 2. It was found that many descriptors, such as park area percentage, the total number of full bathrooms and total number of floors, were positively correlated with the property price, while the distance to transportation and the house age were both negatively correlated. Moreover, for the concerns about the effects of park area percentage and distance to transportation on the housing prices, we specifically portrayed the relationship between house property prices and the GREEN and TRAFFIC features on data visualization presented in Fig 4. These results obtained are in accordance with similar studies reported in the literatures [19, 20, 51, 63–65].

**Table 2:** Correlation coefficients of the house price and each feature.

Variables	(a)	(b)	(c)	(d)	(e)	(f)	(g)	(h)	(i)	(j)	(k)
LAV_TOTAL(a)	1.0000	0.1827	-0.1336	0.4741	0.4159	0.6615	0.2607	0.7329	0.1185	0.3294	-0.4602
LLAND_SF (b)	0.1827	1.0000	-0.0336	0.3420	0.3740	-0.0870	0.1324	0.1249	0.1730	0.0041	0.1919
LAGE (c)	-0.1336	-0.0336	1.0000	0.0011	-0.0838	-0.0593	-0.1173	-0.3947	0.0047	0.1548	0.0836
LR_T_RMS (d)	0.4741	0.3420	0.0011	1.0000	0.8032	0.4598	0.7870	0.5589	-0.2900	0.1475	0.1285
LR_BDRMS (e)	0.4159	0.3740	-0.0838	0.8032	1.0000	0.2645	0.5933	0.4393	-0.2273	-0.0409	0.0918
LN_FLOORS (f)	0.6615	-0.0870	-0.0593	0.4598	0.2645	1.0000	0.4140	0.6887	-0.0265	0.2576	-0.1525
R_KITCH (g)	0.2607	0.1324	-0.1173	0.7870	0.5933	0.4140	1.0000	0.5810	-0.3905	0.0877	0.2306
R_FULL_BTH (h)	0.7329	0.1249	-0.3947	0.5589	0.4393	0.6887	0.5810	1.0000	-0.1402	0.2167	-0.1537
R_HALF_BTH (i)	0.1185	0.1730	0.0047	-0.2900	-0.2273	-0.0265	-0.3905	-0.1402	1.0000	0.0918	-0.1722
GREEN (j)	0.3294	0.0041	0.1548	0.1475	-0.0409	0.2576	0.0877	0.2167	0.0918	1.0000	-0.2938
TRAFFIC (k)	-0.4602	0.1919	0.0836	0.1285	0.0918	-0.1525	0.2306	-0.1537	-0.1722	-0.2938	1.0000

Upon data inclusion in a multivariate regression model of the house transaction values, a hedonic price function of individual feature variables for each city can be generally expressed as

$${LAV}_{TOTAL}=\alpha +\beta_{1}{LLAND}_{SF}+\beta_{2}LAGE+\beta_{3}{LR}_{T\_RMS}+\beta_{4}{LR}_{BDRMS}+\beta_{5}{LN}_{FLOORS}+\beta_{6}R_{KITCH}+\beta_{7}R_{FULL\_BTH}+\beta_{8}{R_{HALF\_BTH}+\beta }_{9}GREEN+\beta_{10}TRAFFIC,$$

where LAV_TOTAL_ was the natural logarithm of total housing prices, α was the intercept term of the regression and *β*_*i*_ with *i* = 1,2,3,…,10 were the estimated coefficients for the independent variables, as described in Table 1. Consequently, based on the transaction datasets, the total housing price in Boston was given by LAV_TOTAL_ = 11.040808+0.090124LLAND_SF_+0.080051*LAGE*+0.315557*LR*_*T_RMS*_+0.230848LR_BDRMS_+0.582316LN_FLOORS_−0.207885R_KITCH_+0.304499*R*_*FULL_BTH*_+0.101733*R*_*HALF_BTH*_+0.007934GREEN−0.000075TRAFFIC.

The MAE, MSE and RMSE were computed to be 0.2056, 0.0624 and 0.2498, respectively. Following the approaches, the hedonic price function corresponding to each city was constructed to compute the statistical error values, as shown in Table 3. Low statistical errors demonstrated the ability of HPM established in this study for the accurate quantitative descriptions of housing prices in Boston, Milwaukee, Tokyo and Taipei, respectively.

**Table 3:** Comparisons of the statistical errors obtained using HPM and LSA models for each city.

City	Model	MAE	MSE	RMSE
**Boston**	HPM	0.2056	0.0624	0.2498
	LSA	0.3372	0.1505	0.3879
**Milwaukee**	HPM	0.3752	0.2564	0.5063
	LSA	0.2047	0.0675	0.2598
**Taipei**	HPM	0.3605	0.2070	0.4550
	LSA	0.1848	0.0861	0.2934
**Tokyo**	HPM	0.6845	0.1997	0.4469
	LSA	0.0797	0.0238	0.1542

**Fig 5:**
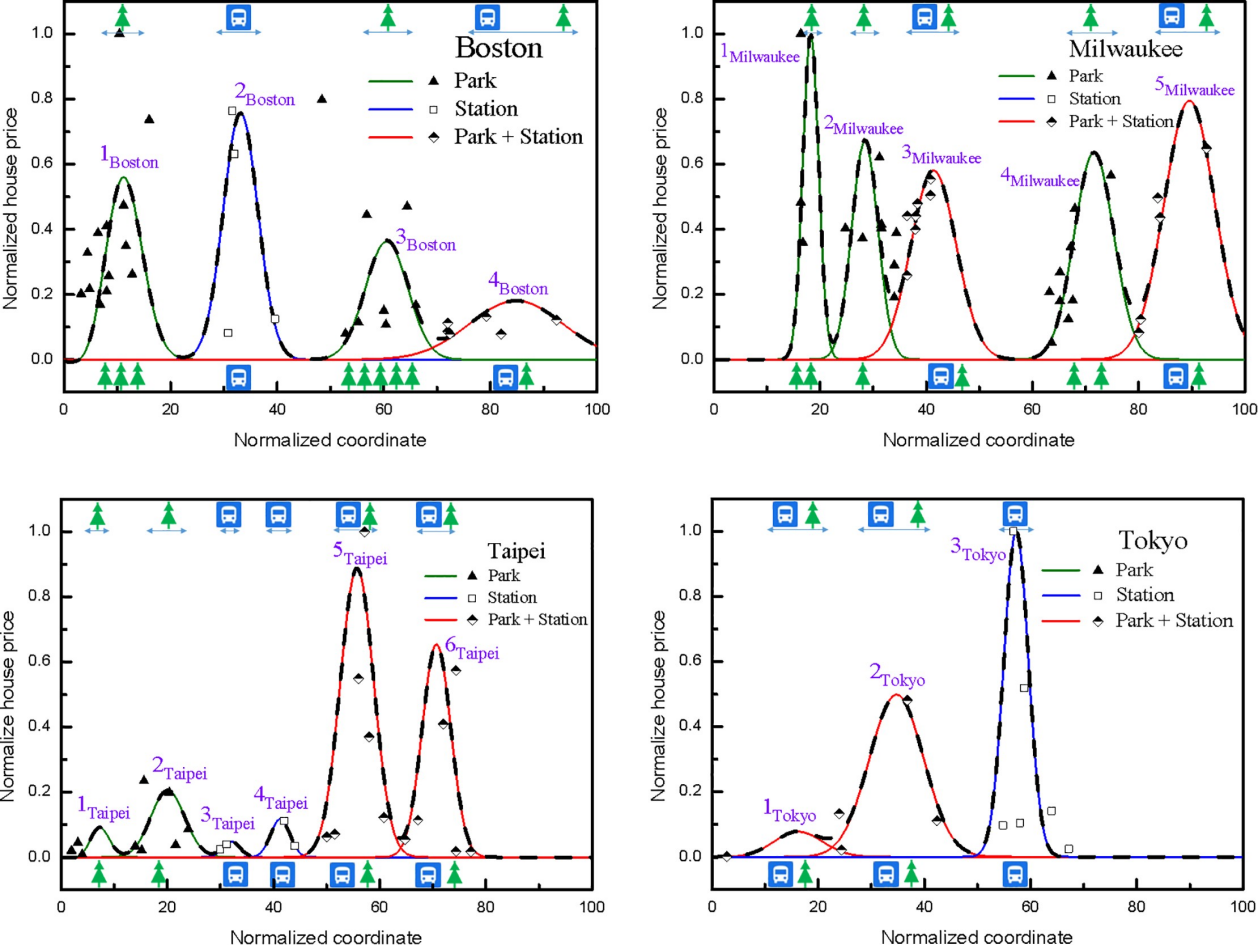
House prices normalized to the maximum value of the (a) Boston; (b) Milwaukee; (c) Taipei and (d) Tokyo for the selected routes presented in Fig 1, respectively. The route distances are indicated on a scale from 0 to 100.

The spatial heterogeneity of infrastructure amenities response to housing prices has exhibited a profound role for property markets. To homogenize data properly and make symmetry analysis more practical and feasible, all collected values in house prices were normalized to the maximum value of the transaction prices for Boston, Milwaukee, Taipei and Tokyo, respectively, while the route distances were indicated on a scale from 0 to 100. Both map legends, including Park and Station appearing in USGS National Map, the relative location coordinate axis which showed data location information point by point were used for visual clarity. This was because of that the residential property values collected along the route given mainly depended on the effects of transportation systems and public parks in this work. Not surprisingly, the profile of the property price volatility along the routes of interest can be understood as a wave-like function of urban housing market system, similar to propagation of the transaction volume-price probability wave or the financial pilot wave [34–36]. Observed data on housing prices from government statistics datasets to depict the variance of the housing prices along designated routes taken from USGS National Map Viewer, as described in Fig 1, for Boston, Milwaukee, Taipei and Tokyo were illustrated in Fig 5, respectively. Significantly, there were four, five, six and three HPW peaks observed for Boston, Milwaukee, Taipei and Tokyo, respectively. By taking account of broadening, symmetry and shift error, solid and dotted lines in Fig 5 corresponded to the Infrastructure-related volatilities and the whole HPWs, respectively. The clear spatial-spectral characteristics undoubtedly indicated that the whole price volatilities composed of individual HPW were modulated by the Park and Station. Mainly owing to the fact that house prices were influenced by many other feature parameters, while remaining a challenge, a discussion about the exact values in terms of linear combination coefficients of the individual HPW was not in the scope of this study. We aimed to extract the symmetry characteristics associated with the impacts of city infrastructure on house prices from public government institute datasets that seem to be highly asymmetric, or even chaotic.

A seven-dimensional Lie algebra was carried out based on the prolongation structure of the Schrödinger equation used to model housing price dynamics in this work, similar to our previous report in drosophila developmental processes [66]. It has been known that there are seven main types of symmetry in the Schrödinger equation, leading to a recursion operator generating an infinite sequence of Lie-Bäcklund symmetries admitted by the Schrödinger equation, as mentioned earlier [61]. Careful inspection of the well-established Schrödinger algebra commutation relations suggested that these two amenity-dependent wave packets were assigned as follows: Lie-Bäcklund infinitesimal generators or symmetry vector fields of Y_2_ and Y_5_ for the urban transportation and public parks, respectively. The change in the intrinsic symmetry properties of the house value was subject to the influence of an external attractive field created by a city infrastructure. The LSA for extreme irregularities in the evolution of prices along the selected routes was expected to be realized by the infrastructure features. A house price of an entirely new building may be individually regarded as a wave-packet in the ground state, denoted as u_0_(x), while the influencing factors of interest were treated as perturbations. The time-independent Schrödinger equation governs the transitions among quantum states corresponding to the physical transaction prices. Being well characterized as exerting positive influences on real estate prices, it was found that infrastructure amenities, in the forms of transportation systems and public parks, intrinsically increased the housing price in larger proportion. As a group-theory approach for housing market dynamics, our data suggested that urban transportation caused a break in the symmetry from invariant solution u_0_(x) to u_1_(x), i.e., 2_Boston_, 3_Taipei_, 4_Taipei_, and 3_Tokyo_, while public parks changed invariant solution u_0_(x) to u_2_(x), i.e., 1_Boston_, 3_Boston_, 1_Milwaukee_, 2_Milwaukee_, 4_Milwaukee_, 1_Taipei_, and 2_Taipei_. Furthermore, effects of the combination of both influencing factors, i.e., transportation and parks, on the house price then stimulated the invariant function to be u_3_(x), i.e., 4_Boston_, 3_Milwaukee_, 5_Milwaukee_, 5_Taipei_, 6_Taipei_, 1_Tokyo_ and 2_Tokyo_. As a result, the governing equations regarding to the mechanism of HPW localization in house markets can be completely expanded by the Lie symmetry vector fields, such as Y_2_ and Y_5_ in the present work. Compared with the statistical results described by the HPM, MAE, MSE and RMSE were used to assess the LSA performances in terms of accuracy, as presented in Table 3. Generally, lower statistical errors of estimation in property price volatilities revealed that the LSA in this study substantially has considerable accuracy, even the lack of detailed modelling of individual socio-economic processes. The connections examined were expected to provide important clues regarding not only the impact of regional housing markets, but also the symmetry-related characteristics in global housing markets.

The full-width half-maximum (FWHM) of the HPW was generally taken as the figure of merit for describing the positive ripple phenomena generated by infrastructures on house prices in the four cities. Accordingly, in an attempt to corroborate the findings of a symmetry role of features of interest in property values, we comprehensively characterized the spatial spillover effect by evaluating the broadenings of the HPW peaks. Acting on the Schrödinger equation with the infinitesimal generators associated with Station or Park, i.e., Y_2_ or Y_5_. Fig 5(A) showed that both 1_Boston_ and 3_Boston_ exhibit broader HPW lines along the selected route in Boston by comparing with the FWHM of the peak 2_Boston._ The difference in wave spreading closely reflects that Park has more spillover effect than Station on housing prices. Furthermore, among the four HPW peaks, the most spreading observed was 4_Boston_ caused both by Station and Park, i.e., Y_2_ and Y_5_. As shown in Fig 5(B)–5(D), the HPWs along the routes illustrated in Fig 1 in Milwaukee, Taipei and Tokyo can also reveal the similar characteristics. Thus, manifestly, two influence factors combined permit more spatial extension of 3_Milwaukee_ and 5_Milwaukee_ relative to 1_Milwaukee_, 2_Milwaukee_ and 4_Milwaukee_. Analogously, the linewidths of 5_Taipei_ and 6_Taipei_ were wider than those of 1_Taipei_ and 2_Taipei_, while 3_Taipei_ and 4_Taipei_ were the narrower lines. Meanwhile, the profiles of 1_Tokyo_ and 2_Tokyo_ are broader than which of 3_Tokyo_. These results obtained from LSA were consonant with the invariant characteristics, as schematically presented in Fig 2, intrinsically corroborating that group action left the set of observables on the financial-related behaviors invariant [37].

Consistent with the abstract algebraic results regarding housing dynamics, the fact that the association between the volatilities in the housing price and the changes in the infrastructure amenity was governed by the principle of symmetry. Implicit in the proposed invariant-theoretical descriptions was the concern that interdisciplinary research and collaboration are permitting a more comprehensive understanding of fluctuating symmetry and asymmetry in the socio-economic dynamics. Nonetheless, as regards the group theoretical aspect, an interesting question which merits attention is that the dataset obtained inherently possessed symmetry properties, while measurements performed subtly correspond to the symmetry transformations or the group actions belonging to the symmetry groups. It is instrumental in the causal interpretations of the functional link between scale invariant characteristics and house price capitalization effects, as will be reported elsewhere.

## Conclusions

Lie symmetry analysis of the official house price dataset at the worldwide level has been successfully performed to assess the roles of urban infrastructures in housing markets, including Boston, Milwaukee, Taipei and Tokyo, mainly focusing on the effects of transportation systems and public parks on residential property values. Albeit the real estate dynamics were complex processes, urban housing market system can be phenomenologically understood as a crystal-like matrix composed by the quantum harmonic potential wells, while the house prices may be regarded as wave-packets. According to the observation of spatial ripple effects on house prices in the four cities investigated, the Schrödinger Lie algebra indeed backs up the hidden fact that the mass transit systems and the public green lands possesses Lie point symmetry Y_2_ and Y_5_, respectively. Compared satistically with MAE, MSE, and RMSE obtained by HPM, the Lie symmetry analysis of the Schrödinger equation approach developed herein has successfully used to elucidate the intrinsic impact of the location-dependent price information with a view not only to be consonant with the observed residential property values of the cities internationally, but also to understanding invariant feature of the housing market for further study. Indeed, it was believed that there is no doubt that symmetry has been one of the most fundamental and fruitful concepts in economist’s attempts to decipher the inner workings of the property price volatilities with the impact of positive and negative attributes.

## Supporting information

S1 Data (XLSX)
